# No Evidence of Experimenter Demand Effects in Three Online Psychology Experiments

**DOI:** 10.1162/OPMI.a.367

**Published:** 2026-07-15

**Authors:** Lucas Woodley, Xavier Roberts-Gaal, Rachel Calcott, Fiery Cushman

**Affiliations:** Department of Psychology, Harvard University, Cambridge, MA, USA

**Keywords:** demand effects, experimenter demand, internal validity, reactance

## Abstract

Experimenter demand effects occur when participants alter their behavior to align with perceived study hypotheses, threatening internal validity. Concern about demand effects is pervasive in psychology. Experimenter demand may be especially acute in studies relying on experienced participants recruited online (e.g., via Prolific), who may readily guess hypotheses, or when using common paradigms (e.g., vignette studies and interventions) where study goals are transparent. We conducted three preregistered experiments (*N* = 2,252) examining whether explicit demand cues influence online participants’ behavior across three paradigms commonly used in psychology: a dictator game, replicating prior work on demand effects (Experiment 1); a moral dilemma vignette (Experiment 2); and an intervention on group attitudes (Experiment 3). We randomly assigned participants on Prolific to receive information about the study’s hypothesis or to a no-information control. As expected, we find that receiving such information significantly shifts participants’ beliefs about the study’s hypothesis, creating the potential for experimenter demand effects. Yet we find no evidence that learning any study’s hypothesis alters participants’ behavior, judgments, or attitudes, suggesting that demand effects may be elusive in online samples. Bayesian region-of-practical-equivalence (ROPE) analyses indicate that experimenter demand effects in these paradigms are typically confined to small magnitudes (|*d*| < 0.20), suggesting that effects larger than Cohen’s *d* = 0.20 are unlikely to be fully explained by experimenter demand alone. These findings offer important insights for the design and interpretation of modern online psychology experiments.

## INTRODUCTION

Experimenter demand effects—changes in participant behavior triggered by cues as to the study’s purpose or the participant’s expected role—are a classic concern in social science, mentioned in textbooks (e.g., Nichols & Edlund, 2023; Schacter et al., 2021) and hundreds of articles in top journals.^1^ If participants act as “good subjects” in an experiment, observed results may reflect what participants think the researcher wants rather than true effects of experimental manipulations, threatening internal validity (Orne, 1962; Zizzo, 2010). 

Do demand effects menace experiments conducted on large, online platforms with methods commonly used by modern psychologists? Two major shifts in modern psychology motivate the present research. First, psychologists have shifted from conducting in-person laboratory experiments to running studies online using large crowdwork platforms. In laboratory experiments, demand can arise from subtle behavior or context cues (e.g., experimenter demeanor, recruitment materials, instructions, laboratory environment) that signal desired behavior to participants (Orne, 1962; Sigall et al., 1970). However, modern psychology experiments often occur online, where participants have minimal face-to-face contact with researchers. One might therefore wonder whether the risk of demand effects is lower in online studies. Alternatively, online participants from convenience platforms are often experienced survey-takers; these participants may be highly adept at guessing hypotheses, thus increasing the risk of demand effects. Participants on these platforms must also maintain high approval ratings (Berinsky et al., 2014) and may worry that researchers will reject their submitted data (imposing financial and reputational costs) if they do not conform to experimenters’ expectations.

Second, tasks and survey designs used by psychologists today differ considerably from those used in classic studies, presenting a different set of demand-related challenges. Contemporary studies often use highly-standardized, text-based vignettes or games that experienced participants may have previously encountered. Within-subjects designs can make condition contrasts salient (Hsee, 1996), and routine quality controls (e.g., comprehension checks) can highlight the construct under study. Outcomes are often collected immediately after interventions using face-valid items, with few filler tasks to obscure hypotheses. In contrast to many classic laboratory studies that relied on idiosyncratic props, confederates, and extended cover stories, these standardized, repeated, and tightly coupled procedures create new avenues for experimenter demand.

Prior work examining demand effects offers mixed evidence. A recent meta-analysis of mostly in-person, student samples finds that manipulating participants’ beliefs about the study hypothesis tends to produce a small but significant increase in hypothesis-consistent responding (Coles et al., 2025). Demand effects were also observed to be heterogeneous across study contexts: Between-subjects manipulations of demand characteristics prompted larger demand effects than within-subjects designs, and demand effects were smaller in online (versus in-person) studies (Coles et al., 2025). However, most studies of demand effects were conducted prior to the popularization of online survey platforms in behavioral research (Anderson et al., 2019; for an overview, see Coles et al., 2025).

Recent examinations of demand effects among Amazon Mechanical Turk (MTurk) participants find evidence of small though inconsistent demand effects in economic games (de Quidt et al., 2018; Winichakul et al., 2024) and survey experiments used in political science (Mummolo & Peterson, 2019). Specifically, de Quidt et al. (2018) provided MTurk participants with explicit information about what behavior was expected (e.g., giving more or less money than one normally would in a dictator game). Across multiple economic games, they found that such instructions had either a modest (∼0.13 *SD* effect size) or a statistically insignificant effect on participants’ behavior (9 out of 11 games yielded null results). Only strong direct demand cues (i.e., “You will do us a favor if you do X”) consistently shifted participants’ behavior across games (0.23 to 1.06 *SD* effect size; de Quidt et al., 2018). However, Winichakul et al. (2024) applied the same strong direct demand cues in four other economic tasks involving lotteries and charitable donations and observed inconsistent, quantitatively small, and qualitatively irrelevant effects in laboratory, Prolific, and MTurk samples. Similarly, Mummolo and Peterson (2019) replicated five well-known political-science survey experiments and randomly revealed each study’s hypothesis to participants. They found that neither subtle nor explicit cues about the study’s hypothesis affected the manipulation’s average treatment effect (Mummolo & Peterson, 2019).

If anything, researchers have shown that experimenter demand can backfire, inducing psychological reactance. One paper reports that Prolific participants who habitually play violent video games downplay their aggression when they believe researchers are trying to prove that violent video games and aggression are linked (Seetahul & Greitemeyer, 2024). This paper studied a salient political topic in which participants were personally invested, perhaps the setting most conducive to experimenter effects (Corneille & Lush, 2023), but atypical of many psychology experiments investigating basic social or cognitive mechanisms.

Nevertheless, the spectre of experimenter demand continues to haunt psychology. One recent paper in the leadership literature argues that certain priming interventions manipulate MTurk participants’ beliefs about experimental hypotheses, creating the “potential for demand” (Khademi et al., 2021). Another recent paper in the emotion literature argues that measures collected immediately after a seemingly-related affect induction procedure are subject to demand effects (Wenzel et al., 2024). But critically, their manipulation did not affect participants’ beliefs about the study’s hypothesis.

In sum, prior work has mostly focused on in-person studies, relies on a limited set of paradigms, and has yielded mixed results. Demand effects have been consistently observed only in small, in-person student samples and when demand cues are especially pronounced. This leaves researchers using large, online samples without clarity concerning the risks of demand.

**Figure 1.**
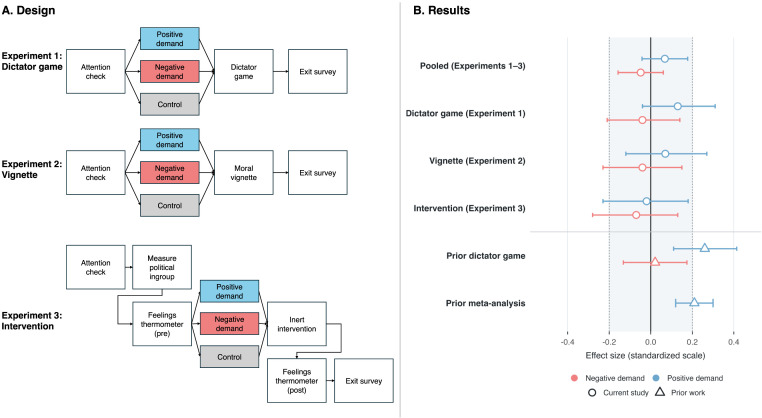
Summary of design and results from our three studies. (A. Design) Each study used a similar experimental design, measuring both positive and negative demand in an online experiment, with three commonly used task types (dictator game, vignette, intervention). Our experiments had ns ≈ 250 per cell. (B. Results) Observed demand effects were statistically indistinguishable from zero. The plot shows means and 95% confidence intervals for standardized mean differences derived from frequentist analyses of each experiment and an inverse variance-weighted fixed-effect estimator pooling all experiments (circles). Prior measurements of experimenter demand from a previous dictator game experiment (de Quidt et al., 2018; standardized mean difference from regression coefficient) and a meta-analysis primarily including small-sample, in-person studies (Coles et al., 2025; Hedges’ *g* statistic) are also shown for comparison (triangles). The main text includes Bayesian analyses that quantify our uncertainty over the size and direction of demand effects.

In the present work, we aim to provide increased clarity by directly testing whether revealing the study’s hypothesis alters behavior among experienced Prolific participants. This follows Orne’s (1962) classical definition of experimenter demand as participants’ efforts to infer and conform to perceived experimental hypotheses, though we recognize that “demand effects” are sometimes used to refer to any cues that may bias experimental results. We focus on three paradigms that exemplify those commonly employed in modern social and cognitive psychology experiments: a dictator game, a moral dilemma vignette study, and an intervention on group attitudes. In each study, our strategy is to allocate participants between-subjects to demand cues (testing both positive and negative demand cues against a neutral control, Figure 1A), measure their beliefs about experimental hypotheses (establishing experimenter demand), and quantify the size of demand effects (Figure 1B).

Crucially, we disentangle awareness of the hypothesis (experimenter demand) from its downstream behavioral effects, extending prior work which found that participants’ beliefs about the purpose of the study determined whether an effect emerges (Durgin et al., 2009; Firestone & Scholl, 2016). By isolating these mechanisms, our design clarifies whether participants’ awareness of the hypothesis is sufficient to produce behavioral effects. In our preregistered hypotheses, we anticipated that positive demand cues would shift behavior in the predicted direction (e.g., greater giving in the dictator game), while negative demand cues would produce the opposite pattern, yielding effects of 0.1–0.3 *SD* (following de Quidt et al., 2018). We further predicted that participants would accurately infer each study’s hypothesis from the demand cues and that the magnitudes of positive and negative demand effects would be similar. Contrary to our expectations, we found that while demand cues strongly influenced participants’ *beliefs* about each study’s hypothesis, they did not significantly alter their behavior, judgments, or attitudes. We provide a summary of our estimates of demand effect magnitudes across studies in Table 1.

**Table 1.** Estimated size of demand effects, across studies, using frequentist regression techniques.

**Task**	**Response variable**	**Demand direction**	**Unstandardized**	**Standardized**	
			**Our study**	**Our study** ^**1**^	**Previous literature**
Dictator game	0 to 100-cent allocation	+	3.39 [−1.07, 7.84] cents	0.13 [−0.04, 0.31]	0.26 [0.11, 0.41]^4^
		−	−0.91 [−5.35, 3.54] cents	−0.04 [−0.21, 0.14]	0.02 [−0.13, 0.17]
Moral vignette	Yes/No judgment	+	*OR*: 1.14 [0.80, 1.62]	0.07 [−0.12, 0.27]^2^	
		−	*OR*: 0.93 [0.66, 1.32]	−0.04 [−0.23, 0.15]	
Intervention	Change score, 100-pt feeling thermometer	+	−0.11 [−1.02, 0.80] pts	−0.02 [−0.23, 0.18]	
		−	−0.33 [−1.24, 0.58] pts	−0.07 [−0.28, 0.13]	
***Overall***	Pooled effect size	+		0.07 [−0.05, 0.17]^3^	0.21 [0.12, 0.30]^5^
		−		−0.05 [−0.16, 0.06]	

^1^ Regression coefficient and 95% confidence interval with indicator predictor and standardized response variable. Standardization computed using control condition mean/*SD* (cf. de Quidt et al., 2018).

^2^ We convert from an odds ratio to a linear scale using Chinn’s method (Chinn, 2000).

^3^ Regression coefficient of a frequentist fixed-effect estimator using inverse variance weighting.

^4^ From the weak demand condition in de Quidt et al. (2018). Regression coefficient with an indicator predictor and standardized response variable.

^5^ Hedges’ *g* statistic from Coles et al. (2025).

## EXPERIMENT 1: DICTATOR GAME

In Experiment 1, we conducted a direct replication of the dictator game (weak demand condition) used in de Quidt et al. (2018), as it was among the only economic games that showed clear evidence of demand. We used the materials from de Quidt and colleagues’ replication package and assigned participants to one of three conditions: a Positive Demand condition wherein participants received a demand cue indicating that experimenters hypothesized participants would *increase* their response, a Negative Demand condition wherein participants received a demand cue indicating that experimenters hypothesized participants would *decrease* their response, and a Control condition without a demand cue. This design allows us to test whether participants alter their responses in the direction of the demand cue. Importantly, we then measured participants’ beliefs regarding the study’s hypothesis. This lets us separate two components of demand effects: (1) whether the demand manipulation successfully changed participants’ *beliefs* about the hypothesis, and (2) whether it changed their *responses*.

### Methods

#### Participants.

All participants were recruited from Prolific on October 23, 2024, and were paid at a rate of $15/hour. Participants were English-speaking U.S. adults. Following prior work (de Quidt et al., 2018), we recruited *N* = 751 participants (approximately 250 per condition) and restricted eligibility to participants with more than 500 tasks completed. We also restricted eligibility to participants with high prior approval ratings (>99%), though we note that applying alternative approval rate requirements (e.g., >95%) are unlikely to change the sample that is recruited because Prolific removes most participants with approval rates lower than 97% (Gordon, 2025).

#### Procedure.

Participants first answered an instructional manipulation (attention) question and then were instructed to allocate $1 between themselves and another randomly selected Prolific participant. Participants were informed that the task involved real money and that whatever they did not give, they would keep for themselves. Following de Quidt et al. (2018), participants in the Positive Demand condition received a demand cue intended to increase the amount of money they gave to the other participant (“We expect that participants who are shown these instructions will give more to the other participant than they normally would.”). Participants in the Negative Demand condition received a demand cue intended to decrease the amount of money they gave to the other participant (“We expect that participants who are shown these instructions will give less to the other participant than they normally would.”). Participants in the Control condition received no additional information. All participants then allocated $1. After allocating $1, participants were asked a dichotomous question about what they thought was the research study’s hypothesis (“What do you think was the hypothesis of this research study?”—Participants would send a large/small share of the $1). To confirm understanding of the task, we also asked participants whether or not their decision involved real money. Participants then provided demographic information and answered additional exploratory measures (not reported here but available in the dataset posted to OSF).

### Results and Discussion

We report frequentist and Bayesian regression analyses modelling participants’ beliefs about the study hypothesis (1 = *large share sent*, 0 = *small share sent*) and allocations ($0.00–$1.00).

As expected, we find that providing demand cues strongly affected participants’ beliefs about the study’s hypothesis. A binomial logit regression shows that positive demand cues significantly increased participants’ likelihood of believing the study’s hypothesis was about sending a large share of the $1 to the other participant (*B* = 2.08, *SE* = 0.22, *z* = 9.35, *p* < .001, *p*_BH_ < .001^2^), whereas negative demand cues significantly decreased this likelihood (*B* = −0.75, *SE* = 0.31, *z* = −2.46, *p* = .014, *p*_BH_ = .047) relative to control. In other words, 57% of participants in the Positive Demand condition (141 out of 249) believed that the study’s hypothesis was that participants would send a large share of the $1 to the other participant, whereas only 7% of participants in the Negative Demand condition (18 out of 252) and 14% of participants in the Control condition (35 out of 250) did so.

**Figure 2.**
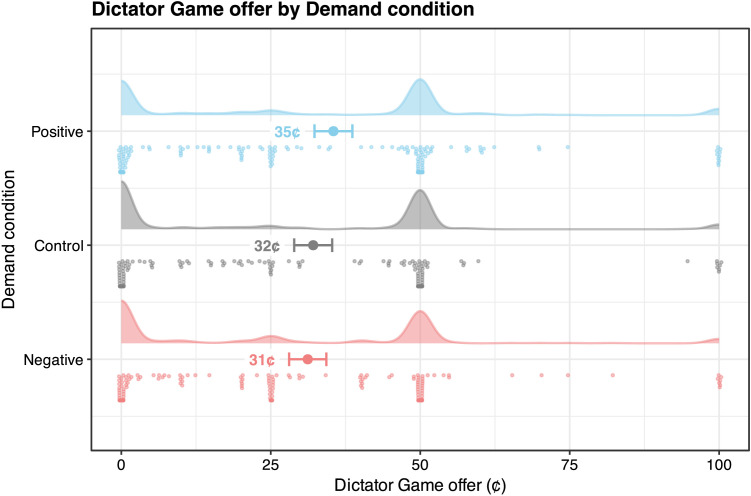
Experiment 1 results. Each row plots the mean and 95% confidence interval (middle), distribution (top), and raw data (bottom) of offers in the dictator game by demand condition (positive = sky blue, negative = coral, control = grey). Most participants offered below 50 cents. The means were not statistically distinguishable across conditions.

Yet, critically, these demand cues had no significant effect on participants’ allocation decisions, as can be seen in Figure 2. A linear regression shows that participants gave similar amounts of money in the Positive Demand (*B* = 0.034, *SE* = 0.023, *t* = 1.49, *p* = .136, *p*_BH_ = .339) and Negative Demand conditions (*B* = −0.009, *SE* = 0.023, *t* = −0.40, *p* = .688, *p*_BH_ = .688) relative to control. Put differently, participants in the Positive Demand condition gave M = $0.35 (*SD* = $0.25), whereas participants in the Negative Demand condition gave M = $0.31 (*SD* = $0.25) and participants in the Control condition gave M = $0.32 (*SD* = $0.25). Using a standardized measure of allocation following de Quidt et al. (2018), we also find that demand effects are statistically insignificant and small, with positive demand cues increasing giving by 0.13 *SD* (*p* = .136, *p*_BH_ = .271) and negative demand cues reducing giving by 0.04 *SD* (*p* = .688, *p*_BH_ = .688) relative to control. We do not detect a difference in the effects of positive and negative demand (Wald *χ*^2^(1) = 3.60, *p* = .058, *p*_BH_ = .144). These patterns persist when controlling for participants’ attentiveness, as there were no significant effects of positive demand cues (*B* = −0.16, *SE* = 0.32, *t* = −0.51, *p* = .611, *p*_BH_ = .679) or negative demand cues (*B* = −0.28, *SE* = 0.35, *t* = −0.79, *p* = .427, *p*_BH_ = .558) on allocations, nor were there significant interaction effects of condition and attentiveness (Attention × Positive Demand: *B* = 0.32, *SE* = 0.33, *t* = 0.98, *p* = .327, *p*_BH_ = .545, Attention × Negative Demand: *B* = 0.28, *SE* = 0.36, *t* = 0.76, *p* = .446, *p*_BH_ = .558). Table 1 reports all coefficients for models using standardized and unstandardized measures.

We obtained similar results within a Bayesian framework using a binomial logit regression and an ordered beta regression. Compared to the Control condition, positive demand cues significantly increased participants’ likelihood of believing the study’s hypothesis was about sending a large share of the $1 to the other participant (*B* = 2.09, 95% equal-tailed Credible Interval (CrI) [1.66, 2.54]), whereas negative demand cues significantly decreased this likelihood (*B* = −0.77, 95% CrI [−1.40, −0.17]).

As in our frequentist analyses, however, demand cues did not significantly affect participants’ allocations. Compared to the Control condition, positive demand cues did not have a significant effect on participants’ allocations (*B* = 0.02, 95% CrI = [−0.13, 0.18], posterior probability [*B* > 0] = 0.62), as did negative demand cues (*B* = −0.07, 95% CrI = [−0.22, 0.08], posterior probability [*B* < 0] = 0.81), indicating at most weak evidence for demand effects. We also did not find evidence for a difference in magnitude of demand effect by direction (posterior median |*B*_Positive_| − |*B*_Negative_| = −0.02, 95% CrI = [−0.18, 0.11], posterior probability [|*B*_Positive_| > |*B*_Negative_| = 0.40]).

Collectively, these results provide evidence that demand cues successfully manipulated participants’ beliefs about the study’s hypothesis. However, we find no significant evidence that inducing such experimenter demand altered participants’ behavior in the dictator game.

## EXPERIMENT 2: MORAL DILEMMA VIGNETTE STUDY

Experiment 2 examined whether experimenter demand alters participants’ moral judgments in a vignette study. We used a realistic sacrificial dilemma from Körner and Deutsch (2023) and Maier et al. (2026) and, as in Experiment 1, assigned participants to one of three conditions (Positive Demand, Negative Demand, or Control).

### Methods

#### Participants.

All participants were recruited from Prolific on October 31, 2024 and were paid at a rate of $15/hour. Participants were English-speaking U.S. adults. As in Experiment 1, we restricted eligibility to participants with high prior approval ratings (>99%) and with more than 500 tasks completed. We recruited *N* = 752 participants (approximately 250 per condition).

#### Procedure.

We used a similar procedure to Experiment 1. Participants first answered an instructional manipulation (attention) question and then were instructed to consider a scenario in which they are a veterinarian who causes animal suffering to develop cures for diseases. More specifically, participants read the following: “*We will now ask you to consider the following scenario: You are a European veterinarian conducting animal experiments on highly contagious germs. On a daily basis, you cause suffering to animals by infecting them with deadly diseases. You are aware that this work provides important insights that, in the long run, can save many other animals. Furthermore, you are a specialist and because of your expertise you can achieve more than other veterinarians. However, you have become a vet in order to heal animals and you are considering quitting your job. If you quit your job, fewer animals will die in the lab, but there will also be fewer findings about animal diseases. As a result, the development of medicines for healing sick animals will be delayed, leading to many preventable deaths. If you continue your job of infecting animals with diseases for medical research, you will keep causing animal suffering, but your research will probably save the lives of even more animals in the future. You will decide whether or not to quit your job.*”

We used similar demand cues to Experiment 1 (“We expect that participants who are shown these instructions will be more/less likely to quit their job than they normally would.”). Participants in the Control condition received no additional information. All participants then decided whether or not to quit their hypothetical job as a veterinarian. After deciding, participants were asked a dichotomous question about what they thought the research study’s hypothesis was (“What do you think was the hypothesis of this research study?”—The experimenters hypothesized that on average participants would/would not quit their job).

To confirm understanding of the task, we also asked participants whether quitting their job reduces the number of animals who die in the lab from experiments versus from preventable diseases. Participants then provided demographic information and answered an exploratory item measuring their motivation for using Prolific (available in the data posted to OSF but not reported here).

### Results and Discussion

We report frequentist and Bayesian regression analyses modelling participants’ beliefs about the study hypothesis (1 = *most participants would quit*, 0 = *most participants would not quit*) and their own moral judgments (1 = *quit job*, 0 = *do not quit job*).

Replicating Experiment 1, we find that providing demand cues strongly affected participants’ beliefs about the study’s hypothesis. A binomial logit regression shows that positive demand cues significantly increased participants’ likelihood of believing the study’s hypothesis was that most participants would quit their job (*B* = 1.29, *SE* = 0.22, *z* = 5.93, *p* < .001, *p*_BH_ < .001), whereas negative demand cues significantly decreased this likelihood (*B* = −1.20, *SE* = 0.19, *z* = −6.40, *p* < .001, *p*_BH_ < .001) relative to control. Put differently, 85% of participants in the Positive Demand condition (213 out of 251) believed that the study’s hypothesis was that most participants would quit their job, whereas only 32% of participants in the Negative Demand condition (79 out of 250) and 61% of participants in the Control condition (152 out of 251) believed this was the study’s hypothesis.

**Figure 3.**
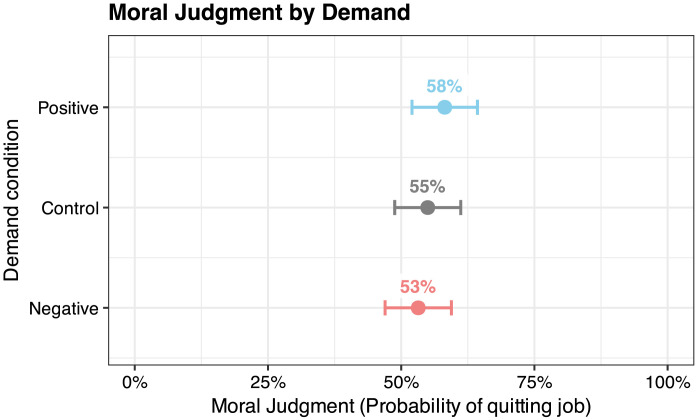
Experiment 2 results. Each row plots the mean and 95% confidence interval of the probability of job-quitting judgments in the moral dilemma by demand condition (positive = sky blue, negative = coral, control = grey). The means were not statistically distinguishable across conditions.

Critically, and replicating the results of Experiment 1, these demand cues had no significant effect on participants’ moral judgments (as can be seen in Figure 3). A binomial logit regression shows that participants’ moral judgments were unaffected in the Positive Demand (*B* = 0.130, *SE* = 0.180, *z* = 0.72, *p* = .473, *p*_BH_ = .669, OR = 1.14 [0.80, 1.62]) and Negative Demand conditions (*B* = −0.072, *SE* = 0.179, *z* = −0.400, *p* = .689, *p*_BH_ = .689, OR = 0.93 [0.66, 1.32]) relative to control. In the Positive Demand condition, 58% of participants (146 out of 251) quit their job compared to 53% of participants in the Negative Demand condition (133 out of 250) and 55% of participants in the Control condition (138 out of 251). An exploratory test of the magnitudes of positive and negative demand effects could not detect a difference (observed |*B*_Positive_| − |*B*_Negative_| = 0.06, permutation test with 10,000 shuffles *p* = .619). These patterns persist in an exploratory model controlling for participants’ attentiveness, as there were no significant effects of positive demand cues (*B* = −0.25, *SE* = 0.59, *z* = −0.42, *p* = .671) or negative demand cues (*B* = −0.51, *SE* = 0.63, *z* = −0.81, *p* = .418) on moral judgments, nor were there significant interaction effects of condition and attentiveness (Attention × Positive Demand: *B* = 0.41, *SE* = 0.62, *z* = 0.67, *p* = .503; Attention × Negative Demand: *B* = 0.47, *SE* = 0.66, *z* = 0.71, *p* = .476).

We obtained similar results within a Bayesian framework using binomial logit regressions. Compared to the Control condition, positive demand cues significantly increased participants’ likelihood of believing the study’s hypothesis was that most participants would quit their job (*B* = 1.30, 95% CrI [0.89, 1.74]), whereas negative demand cues significantly decreased this likelihood (*B* = −1.21, 95% CrI [−1.57, −0.83]).

As in our frequentist analyses, however, demand cues did not significantly affect participants’ moral judgments. Compared to the Control condition, positive demand cues did not have a significant effect on participants’ judgments (*B* = 0.13, 95% CrI = [−0.22, 0.48], posterior probability [*B* > 0] = 0.75), nor did negative demand cues (*B* = −0.07, 95% CrI = [−0.43, 0.28], posterior probability [*B* < 0] = 0.65), indicating at most anecdotal evidence for a true effect of demand cues. We also found at most anecdotal evidence for a difference in magnitude of demand effect by direction (posterior median |*B*_Positive_| − |*B*_Negative_| = 0.02, 95% CrI = [−0.33, 0.33], posterior probability [|*B*_Positive_| > |*B*_Negative_| = 0.56]).

In line with Experiment 1, these results provide evidence that demand cues successfully manipulated participants’ beliefs about the study’s hypothesis. Again, however, we are unable to detect evidence that experimenter demand altered participants’ moral judgments.

## EXPERIMENT 3: INERT INTERVENTION ON GROUP ATTITUDES

**Figure 4.**
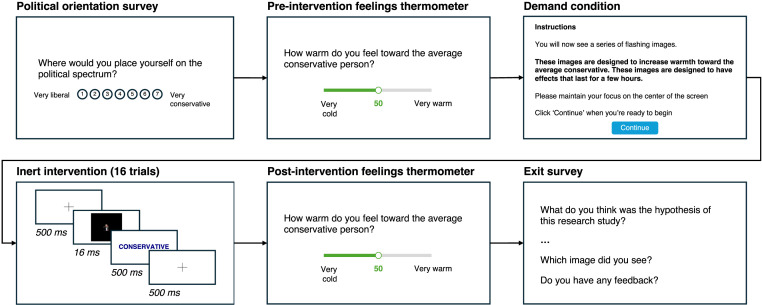
Schematic overview of Experiment 3 procedure. The inert intervention involved sixteen trials. On each trial, participants were presented with a fixation cross for 500 ms, condition-congruent image (100 px max width × 67 px max height) for 16 ms, ideology-congruent word for 500 ms, and fixation cross for 500 ms.

Experiment 3 examined whether experimenter demand alters participants’ attitudes toward social groups, namely their political ingroup, during an intervention study. We designed an intervention on attitudes toward political ingroups that, while superficially plausible, was likely causally inert. Specifically, we used a short visual subliminal priming intervention (see Figure 4 for details) which is highly unlikely to influence attitudes toward political ingroups to a material degree. This design allowed us to isolate demand effects by manipulating experimenter demand (the intervention’s goal was clear) without also manipulating participant attitudes (the intervention was causally inert) or requiring deception (we honestly described the intervention’s goal).

Experiment 3 also used subtler demand cues than did Experiments 1 and 2. Participants were informed that they would see a series of images designed to make them feel greater warmth toward the average ingroup member (Positive Demand condition), less warmth toward the average ingroup member (Negative Demand condition), or not to affect their attitudes toward the average ingroup member (Control condition). These cues were intended to better reflect the information participants might encounter during studies, where overt explicit information about study hypotheses is likely rare.

Moreover, Experiment 3 collected attitudinal information before and after the intervention. This allowed us to explore whether demand cues may elicit a reactance effect, where participants deliberately engage in behavior that counters the perceived hypothesis (Rosenberg & Siegel, 2018); for example, one sign of reactance could be participants in the Positive Demand condition under-reporting warmth toward their political ingroup after the intervention.

### Methods

#### Participants.

All participants were recruited from Prolific on April 10, 2025 and were paid at a rate of $15/hour. Participants were English-speaking U.S. adults. As in Experiments 1 and 2, we restricted eligibility to participants with high prior approval ratings (>99%) and with more than 500 tasks completed. We recruited *N* = 749 participants (approximately 250 per condition).

#### Procedure.

Figure 4 provides an overview of the procedure. Participants were directed to a custom JavaScript application deployed on a Heroku website (leveraging a template from Callaway & Correa, 2025). After consenting, participants answered an instructional manipulation (attention) question and then indicated their political ideology on a 7-point scale (1 = Very Liberal, 7 = Very Conservative). We then collected pre-intervention attitudes towards participants’ political ingroup using a 0–100 feeling thermometer (“How warm do you feel toward the average [liberal / conservative] person?” 0 = Very Cold, 100 = Very Warm). After providing an initial rating, participants were randomly assigned to one of the Positive Demand, Negative Demand, or Control conditions and read the following: “*You will now see a series of flashing images. These images are designed to [increase warmth / decrease warmth / not affect attitudes] toward the average [liberal / conservative]. These images are designed to have effects that last for a few hours. Please maintain your focus on the center of the screen.*”

Participants then completed 16 “subliminal image” trials (see Figure 4). On each trial, participants first viewed a central fixation cross for 500 ms. Then, a centrally presented maximum 100-by-67 pixel condition-congruent intervention image in a 600-by-600 pixel black square appeared for 16 ms (equivalent to approximately one frame on a standard computer monitor with a 60 Hz refresh rate). The intervention image was presented for a shorter duration than many priming studies employ (e.g., two-thirds that of the Very Brief Exposure paradigm; Siegel & Warren, 2025), and covering only a small area of the screen (1/17th that of the Very Brief Exposure paradigm). Finally, the image was replaced with a word mask for 500 ms (political ingroup, i.e., either the word “LIBERAL” or “CONSERVATIVE”), followed by a fixation cross for 500 ms. As we were not focused on estimating participants’ image detection skills, there was no inter-trial interval. So as not to deceive participants, the image we presented was in fact of an ingroup politician (liberal: Barack Obama; conservative: John McCain) shown either smiling (Positive Demand/increase ingroup warmth condition), frowning (Negative Demand/decrease ingroup warmth condition), or with a neutral expression (Control/not affect attitudes condition). A participant was presented with the same image on each trial.

After completing the subliminal image trials, participants then provided post-intervention attitudes towards their ingroup using the same 0–100 feeling thermometer as before. Afterward, participants were asked what they thought the research study’s hypothesis was (“What did you think was the hypothesis of this research study?”—Participants would feel more warmth toward people with shared political views after viewing the images / less warmth toward people with shared political views after viewing the images / participants’ warmth toward people with shared political views would be unchanged after viewing the images).

As an exploratory measure, we also asked participants how they expected the average participant to be affected by the intervention (“How would you expect the feelings of an average participant to be affected by viewing these images?”—More warm / slightly more warm / not affected / slightly less warm / less warm toward the average person in their political group). Because only one participant reported believing the intervention would make others feel “less warm,” we collapsed participants’ responses into three levels (more warm / not affected / less warm). We also asked participants to report their perception of the study’s purpose (“What do you think was the purpose of this study?” To study the relationship between memory and attention / To explore how social norms shape decision-making / To examine the effects of subliminal messages / To analyze how people perceive visual stimuli / To assess the impact of emotions on judgment / To investigate whether experimenter expectations influence participant behavior / To measure political beliefs and attitudes / Other / I’m not sure). And, to check whether participants identified the image they saw, we asked participants to recall which image they saw from an array of 15 images (six true stimuli; six distractor images of Democratic (Joe Biden) and Republican (Donald Trump) politicians making neutral, positive, or negative faces; and three unrelated distractor images). Participants then provided demographic information and answered an exploratory item measuring their motivation for using Prolific (available in the data posted to OSF but not reported here).

### Results and Discussion

We report frequentist and Bayesian regression analyses modelling participants’ beliefs about the study hypothesis (*unchanged* / *more warmth* / *less warmth*) and their own pre-post change in ingroup attitudes (−100 to 100).

Replicating Experiments 1 and 2, we find that providing demand cues strongly affected participants’ beliefs about the study’s hypothesis. A multinomial^3^ logit regression shows that positive demand cues significantly increased participants’ likelihood of believing the study’s hypothesis was that the images would increase ingroup warmth (*B* = 1.01, *SE* = 0.20, *z* = 4.99, *p* < .001, *p*_BH_ < .001). Conversely, negative demand cues significantly increased the likelihood that participants’ believed the study’s hypothesis was that the images would decrease ingroup warmth (*B* = 1.15, *SE* = 0.22, *z* = 5.21, *p* < .001, *p*_BH_ < .001). Put differently, 60% of participants in the Positive Demand condition (151 out of 251) believed that the study’s hypothesis was that the images would increase ingroup warmth, whereas only 25% of participants in the Negative Demand condition (63 out of 254) and 31% of participants in the Control condition (76 out of 244) believed this was the study’s hypothesis. In contrast, 46% of participants in the Negative Demand condition (117 out of 254) believed that the study’s hypothesis was that the images would decrease ingroup warmth, compared to only 8% of participants in the Positive Demand condition (19 out of 251) and 23% of participants in the Control condition (56 out of 244).

**Figure 5.**
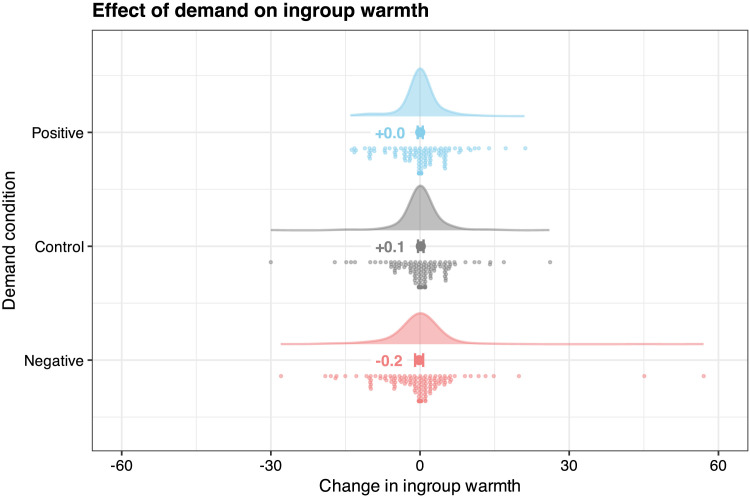
Experiment 3 results. Each row plots the mean and 95% confidence interval (middle), distribution (top), and raw data (bottom) of the change in ingroup attitudes after our inert intervention, by demand condition (positive = sky blue, negative = coral, control = grey). The means were not statistically distinguishable from zero, or from each other.

Critically, and replicating the results of Experiments 1 and 2, these demand cues had no significant effect on participants’ ingroup attitudes (see Figure 5). A linear regression shows that participants’ pre-post change in ingroup warmth in the Positive Demand (*B* = −0.11, *SE* = 0.46, *t* = −0.23, *p* = .817, *p*_BH_ = .817) and Negative Demand conditions (*B* = −0.33, *SE* = 0.46, *t* = −0.71, *p* = .478, *p*_BH_ = .546) were not significantly different from control. In other words, participants’ ingroup warmth in the Positive Demand condition changed by *M* = 0.04 (*SD* = 4.06). This was similar to participants in the Negative Demand condition (*M* = −0.19, *SD* = 6.57) and to participants in the Control condition (*M* = 0.14, *SD* = 4.44). Using a standardized measure of attitude change as in Experiment 1, we also find that demand effects are statistically insignificant and small, with positive demand cues decreasing ingroup warmth by 0.024 *SD* (*p* = .817, *p*_BH_ = .817) and negative demand cues decreasing ingroup warmth by 0.074 *SD* (*p* = .478, *p*_BH_ = .546) relative to control. These patterns persisted in an exploratory model controlling for participants’ attentiveness, as there were no significant effects of positive demand cues (*B* = −0.21, *SE* = 0.44, *t* = −0.47, *p* = .636) or negative demand cues (*B* = −0.17, *SE* = 0.44, *t* = −0.39, *p* = .698) on attitude change, nor were there significant interaction effects of condition and attentiveness (Attention × Positive Demand: *B* = 0.19, *SE* = 0.45, *t* = 0.42, *p* = .675; Attention × Negative Demand: *B* = 0.10, *SE* = 0.45, *t* = 0.22, *p* = .829). As before, an exploratory test of the magnitudes of positive and negative demand effects could not detect a difference (observed |*B*_Positive_| − |*B*_Negative_| = −0.05, permutation test with 10,000 shuffles *p* = .492).

We obtained similar results within a Bayesian framework. Compared to the Control condition, positive demand cues significantly increased participants’ likelihood of believing the study’s hypothesis was that the images would increase ingroup warmth (multinomial logit regression^4^ *B* = 1.02, 95% CrI [0.62, 1.42]), whereas negative demand cues significantly increased the likelihood that participants’ believed the study’s hypothesis was that the images would decrease ingroup warmth (*B* = 1.17, 95% CrI [0.72, 1.62]).

As in our frequentist analyses, however, demand cues did not significantly affect participants’ ingroup attitudes. Compared to the Control condition, positive demand cues had a small, uncertain effect on participants’ attitudes (linear regression *B* = −0.09, 95% CrI = [−1.01, 0.83], posterior probability [*B* > 0] = 0.42), as did negative demand cues (linear regression *B* = −0.32, 95% CrI = [−1.22, 0.58], posterior probability [*B* < 0] = 0.75), indicating at most anecdotal evidence for a true effect of demand cues. We also did not find evidence for a difference in magnitude of demand effect by direction (posterior median |*B*_Positive_| − |*B*_Negative_| = −0.01, 95% CrI = [−0.20, 0.12], posterior probability [|*B*_Positive_| > |*B*_Negative_| = 0.44]).

One possibility is that our results reflect a mix of reactance and demand compliance, or a mixture of a true attitudinal intervention effect and reactance across the population. If so, this heterogeneity may appear as an increased standard deviation in attitude change. As an exploratory analysis, we examined differences in variability in attitude change across conditions using a generalized additive model for location, scale and shape. We find no significant evidence that positive demand cues increased standard deviation in attitude change (*B* = −0.089, *SE* = 0.064, *t* = −1.40, *p* = .163) relative to control. However, we do find that negative demand cues significantly increased standard deviation in attitude change (*B* = 0.392, *SE* = 0.063, *t* = 6.19, *p* < .001) relative to control. A Bayesian generalized additive model for location, scale, and shape likewise revealed no significant evidence that positive demand cues increased standard deviation in attitude change (*B* = −0.091, 95% CrI = [−0.213, 0.026]), but did find that negative demand cues significantly increased such standard deviation (*B* = 0.392, 95% CrI = [0.267, 0.517]).

To further explore whether this increased standard deviation in attitude change reflects a mix of reactance and demand compliance, or of an intervention effect and reactance, we fit mixture models with one to seven components exploring the effect of each demand condition. Overall, the mixture model analysis did not support the existence of latent classes of participants with different response profiles (i.e., compliers and reactors), as mixture component estimates frequently converged on degenerate point-mass solutions and no component in any model revealed a significantly positive or negative effect of demand. We report full details in the Supplemental Materials.

Taken together, these results suggest that the effects of demand cues (and of our intervention) are statistically insignificant. Moreover, we did not observe evidence that demand cues elicited either compliance or reactance in different participants (yielding a mixture of these response profiles at the population level).

Our exploratory analyses also reveal that most participants believed the intervention would not affect others’ attitudes. This was true in the Positive Demand condition (70%, 175 out of 251), the Negative Demand condition (75%, 191 out of 254), and the Control condition (65%, 159 out of 244). We do not find evidence that positive demand cues increased participants’ likelihood of believing the intervention would increase others’ ingroup warmth (multinomial logit regression: *B* = 0.001, *SE* = 0.211, *p* = .996), nor do we find evidence that negative demand cues increased participants’ likelihood of believing the intervention would decrease others’ ingroup warmth (*B* = −0.446, *SE* = 0.316, *p* = .159). When prompted, a small minority of participants were able to correctly guess the study’s true purpose of measuring experimenter effects in each condition (Positive Demand: 18.3%, 46 out of 251; Negative Demand: 23.6%, 60 out of 254; Control: 12.3%, 30 out of 244), though this did not affect their behavior (no significant interaction effects in multinomial logit regression: Guess Correctly × Positive Demand: *B* = 0.10, *SE* = 0.30, *t* = 0.32, *p* = .747; Guess Correctly × Negative Demand: *B* = −0.12, *SE* = 0.28, *t* = −0.43, *p* = .670). Moreover, only 4.01% of participants (30 out of 749, about one in 25) correctly identified the image they were presented with from an array of 15 (see Table S2).

Conceptually replicating and extending Experiments 1 and 2, these results collectively provide evidence that demand cues successfully manipulated participants’ beliefs about the study’s hypothesis in an intervention design. However, consistent with Experiments 1 and 2, we find no significant evidence that inducing such experimenter demand altered participants’ ingroup attitudes. We also find no statistically significant evidence of reactance.

## BOUNDING EXPERIMENTER DEMAND EFFECTS

In each experiment, following prior literature, we expected to observe experimenter demand effects. But although we successfully manipulated participants’ beliefs about each study’s hypotheses, we did not observe evidence that inducing such experimenter demand significantly altered participants’ behavior, judgments, or attitudes. However, we recognize that absence of evidence is not equivalent to evidence of absence. We therefore conducted exploratory analyses aimed at assessing the extent to which our data can help bound the plausible magnitude of experimenter demand effects within online experiments conducted with experienced participants. Specifically, we conducted a Bayesian region-of-practical-equivalence (ROPE) analysis. We note that although our studies use fairly large samples by contemporary standards (approximately *N* = 250 per cell), they are somewhat underpowered for equivalence testing. We therefore pool across studies to estimate the standardized effects of positive and negative demand relative to control.^5^ We specified a region of practical equivalence of Cohen’s *d* = ±0.20, corresponding to a traditionally “small” effect (Cohen, 1988). The posterior mean for the difference between positive demand and control was *d* = 0.04, 95% CrI [−0.05, 0.13]. The posterior mean for the difference between negative demand and control was *d* = −0.04, 95% CrI [−0.13, 0.05]. For both contrasts, 99.98% of the posterior probability mass fell within the ROPE. For practical purposes, our data indicate that effects of experimenter demand in these paradigms with online samples are unlikely to be larger than Cohen’s *d* of ±0.20.

In addition, we ran a frequentist two-one-sided test (TOST) procedure to test for the absence of a meaningful effect of demand (Lakens, 2017). That is, we tested whether our standardized estimates of demand effects, pooled across studies within direction, were within equivalence bounds of *d* = 0.20 (i.e., a “small” effect size). For both directions, the equivalence test was significant (Positive: *z* = −2.15, *p* = 0.016; Negative: *z* = 2.72, *p* = 0.003) and the null hypothesis test was non-significant (Positive: *z* = 1.41, *p* = 0.157; Negative: *z* = −0.87, *p* = 0.383); we can rule out experimenter demand effects larger than |*d*| = 0.2 in our data.

## GENERAL DISCUSSION

Demand effects are frequently cited as a potential hazard in psychology experiments. We conducted three preregistered experiments examining whether explicit cues about a study’s hypothesis produce detectable demand effects in samples of online participants recruited via Prolific. In all three experiments, the experimenter demand manipulations successfully affected participants’ *beliefs* about the study hypotheses, thereby creating the potential for participants to alter their behavior in line with the experimenter’s expectations (or react against them). Critically, however, experimenter demand did not lead participants to produce hypothesis-supporting (or hypothesis-refuting) *behavior*, *judgments*, or *attitudes*. Put simply, all three studies failed to elicit any detectable demand effects in participants’ responses. This pattern persisted across a dictator game measuring economic behaviors (Experiment 1), a vignette study examining moral judgments (Experiment 2), and an intervention on group-level attitudes (Experiment 3). This failure to alter participants’ behaviors, judgments, or attitudes despite inducing demand suggests that demand effects may be weaker than previously thought or highly elusive in online research contexts using modern methods. Notably, because we did not detect change in *either direction*—hypothesis-consistent or hypothesis-inconsistent—these experiments also suggest that effects of reactance to experimenter demand may be similarly weak or elusive.

Our findings strengthen the view emerging in recent literature arguing that simple disclosure of a study’s expected result seldom contaminates data quality in online behavioral research using modern paradigms (Coles et al., 2025; de Quidt et al., 2019; Mummolo & Peterson, 2019; Winichakul et al., 2024). Why might participants register a cue but decline to act on it? One possibility is insufficient motivation: with full anonymity, modest stakes, and no interpersonal rapport with the experimenter, Prolific participants may feel little incentive to “help” the researcher beyond meeting basic task requirements. Additionally, economic generosity, moral judgments, and group attitudes may be governed by stable preferences that mere knowledge of a study’s hypothesis cannot easily override. In Experiment 3, our exploratory analyses are inconsistent with the possibility that different participants demonstrated either compliance with or reactance to experimenter demand cues, yielding a mixture of such responses across the population. Thus, even if rare individuals may treat the cue as a signal to conform (or rebel), their influence on aggregate estimates is insignificant.

Our findings are subject to several limitations. First, our cues stated an expected direction of behavior but offered no social evaluation, financial leverage, or moral pressure. More forceful manipulations (e.g., “You will help us if …” or financial bonuses for hypothesis-consistent behaviors) could elicit larger shifts in participants’ responses (Corneille & Lush, 2023; de Quidt et al., 2018), though manipulations like these are atypical in modern experimental psychology.

Second, our cues were explicit. Perhaps “demand characteristics may be less effective or even have a paradoxical action if they are too obvious” (Orne, 2009, p. 116). Related, it is possible that explicit cues induce a form of experimenter demand (e.g., about how participants should respond to learning hypotheses) unlike those typically encountered in psychology experiments. However, we observe neither demand effects nor paradoxical actions (i.e, reactance), and we test explicit cues that are both very obvious (Experiments 1 and 2) and more subtle (Experiment 3). It remains possible that only minimally obvious cues (e.g., researcher facial expressions or even more subtle messages requiring interpretation by participants) will induce traditional demand effects (cf. Coles et al., 2025; Corneille & Lush, 2023). If so, the field requires a theory of how participants’ interpretation of these minimally obvious demand cues translates into biased estimates.

Third, participants in all three studies may have held clear views on our domains of generosity, animal welfare, and partisan identity. Attitude-neutral or ambiguous tasks (e.g., abstract puzzles, perceptual judgments) may be more strongly susceptible to experimenter demand effects (Firestone & Scholl, 2016). (However, we also do not observe reactance, which we might have expected to occur if participants held clear views contrary to our experimental manipulations; Seetahul & Greitemeyer, 2024.)

Fourth, our studies recruited a US-based population. Other populations may differ in their response to experimenter demand; for example, acquiescence, a related effect, appears stronger in collectivistic populations (Johnson et al., 2005) and in traditional societies (Javeline, 1999).

Beyond these limitations, we note that our sample consisted of Prolific participants who were highly experienced survey-takers. It is possible that novice populations may perceive greater pressure to comply with experimenter’s expectations. The fact that our findings derive from seasoned Prolific participants may count in favor of drawing from this pool for online studies, though researchers may have valid reasons not to do so (e.g., research questions for which participant naïveté is a requirement).

We do not claim that demand effects *never* matter. We assume they sometimes do. Indeed, we originally set out to validate a method for reducing demand effects, only to discover that we could not detect demand effects in the first place. Our findings suggest instead that in the context of typical online psychology experiments with convenience samples, demand effects do not seem to significantly bias participants’ responses overall. Methodologically, these findings are encouraging. Despite the growth of highly experienced online samples, participants’ responses appear to be robust to experimenter demand cues. Across three preregistered experiments, participants reliably detected cues about the researcher’s hypothesis, yet this awareness did not alter their economic choices, moral judgments, or group attitudes. For online psychology studies using standard economic games, moral vignettes, or attitudinal interventions, experimenter demand effects appear—at least in their simplest form—to be more phantom than menace.

## ACKNOWLEDGMENTS

We thank members of the Laboratory for Social Cognitive Science for their guidance. We are grateful for comments we received on an earlier version of this manuscript, including from the reviewers and from Olivier Corneille.

## FUNDING INFORMATION

LW is supported by the NSF GRFP (2140743). XRG is supported by the Department of Defense through the National Defense Science and Engineering Graduate Fellowship Program. This material is based upon work supported by the Air Force Office of Scientific Research under award number FA9550-23-F-0014.

## AUTHOR CONTRIBUTIONS

L.W.: Conceptualization; Formal analysis; Investigation; Methodology; Software; Visualization; Writing – original draft; Writing – review & editing. X.R.-G.: Conceptualization; Formal analysis; Investigation; Methodology. Software; Visualization; Writing – original draft; Writing – review & editing. R.C.: Conceptualization; Formal analysis; Investigation; Methodology; Software; Visualization; Writing – original draft; Writing – review & editing. F.C.: Conceptualization; Funding acquisition; Methodology; Supervision; Writing – review & editing.

## DATA AVAILABILITY STATEMENT

Preregistrations, data, code, and materials are available on an OSF repository (https://osf.io/rkvbd/overview?view_only=2c725a6939ef490d836d9550c19dd309) and AsPredicted (https://researchbox.org/4593).

## TRANSPARENCY AND OPENNESS

This research was approved by the Harvard University-Area IRB (protocol #IRB14-2016). For all studies, the design, sample size, hypotheses, and analysis plan were preregistered: https://researchbox.org/4593. Materials, preregistration forms, de-identified data, and analysis scripts for all studies are available in an Open Science Framework (OSF) repository: https://osf.io/rkvbd/overview?view_only=2c725a6939ef490d836d9550c19dd309. We report how we determined sample sizes, all data exclusions (if any), all manipulations, and all measures in the studies. All analyses were conducted using R statistical software (R Core Team, 2025). Sample sizes were determined using power analyses based on previous research. No artificial intelligence technologies were used in writing this paper. The authors used AI to assist with programming. The authors have thoroughly checked and take responsibility for all lines of code. For details, see the Supplemental Material.

## STATEMENT OF RELEVANCE

Researchers in the social sciences often use online behavioral experiments to test hypotheses. But, if participants in these experiments learn about researchers’ hypotheses, they may change their behavior—intentionally or unintentionally. These *demand effects* can threaten the validity of experiments. Modern social-science experiments frequently rely on experienced participants recruited on large, online platforms (e.g., Prolific) who may be adept at guessing hypotheses. Also, experimenters often run studies that have transparent goals. We ran three online experiments reflecting standard techniques commonly employed in psychology (an economic game, a vignette study about moral judgments, and an intervention on group attitudes), and in each, we measured the effects of revealing our hypotheses to participants. Participants in all three experiments correctly inferred our hypotheses, but did not appear to alter their behavior, judgments, or attitudes. These findings suggest that, despite the prevalence of concerns about demand effects in the literature, experimenter demand is unlikely to threaten the validity of online studies using similar techniques.

## Notes

^1^ A Google Scholar search of publications in top journals—*Nature*, *Science*, *PNAS*, *Annual Reviews*, *Psychological Science*, and American Psychological Association-published journals—yielded 592 papers mentioning demand effects. Details in Supplemental Materials.^2^ Following de Quidt et al. (2018), for our preregistered replication analyses we additionally report *p*-values corrected for a false discovery rate at the *α* = 0.05 level, using the Benjamini-Hochberg method, as *p*_BH_.^3^ We preregistered binomial logit regressions to test our hypotheses that the demand manipulation would affect participants’ beliefs. But because there were three possible directions in Study 3 (increase warmth, decrease warmth, and leave warmth unchanged), a single binomial logit regression is unable to test these hypotheses. The correct test, which we report here, is a multinomial logit regression. This deviation does not alter the conservatism of our test. We report the other alternative analyses that could have answered this question (i.e., independent binomial logit regressions for each condition) in the Supplemental Materials; results do not change.^4^ Same deviation as before; see Supplemental Materials for robustness checks—results do not change.^5^ As above, for Study 2 we derive the standardized mean difference using Chinn’s method.

## Supplementary Material


