# Supplementary material for: No Evidence of Experimenter Demand Effects in Three Online Psychology Experiments
Source: Open Mind (Camb). 2026 Jul 15;10:998–1016. doi: 10.1162/OPMI.a.367 (PMC13421554; doi:10.1162/OPMI.a.367)

**SUPPLEMENTAL MATERIAL**

**for**

**No Evidence of Experimenter Demand Effects in Three Online Psychology Experiments**

**All Studies**

**Research Transparency**

All data and code are available on OSF: https://osf.io/rkvbd/?view_only=4506d1379ec248a989b617529af22916

All preregistration documents are available on AsPredicted: https://researchbox.org/4593&PEER_REVIEW_passcode=LVDSED

**Google Scholar search**

We conducted a search on Google Scholar on August 28, 2025, using the following search terms: ("demand effect" OR "demand characteristic" OR "experimenter expectancy effect") (site:nature.com OR site:apa.org OR site:science.org OR site:psychologicalscience.org OR site:pnas.org OR site:annualreviews.org)

**Bayesian Modeling Details**

We fit all Bayesian models in the R package brms. For each model, we ran four chains with 1,000 warmup steps and 1,000 sampling steps per chain. We used default, uninformative priors provided in brms; these were:

*Ordered beta regression* (Dictator game allocation ~ Positive Demand + Negative Demand)

| **Parameter name** | **Prior** |
| --- | --- |
| b | normal(0,5) |
| b_NegativeDemand | normal(0,5) |
| b_PositiveDemand | normal(0,5) |
| cutone | induced_dirichlet([1,1,1]’, 0, 2, cutzero, cutone) |
| cutzero | induced_dirichlet([1,1,1]’, 0, 1, cutzero, cutone) |
| Intercept | student_t(3, 0.5, 2.5) |
| phi | exponential(0.1) |

*All binomial logit models* (e.g., Belief ~ Positive Demand + Negative Demand)

| **Parameter name** | **Prior** |
| --- | --- |
| b | flat |
| b_NegativeDemand | flat |
| b_PositiveDemand | flat |
| Intercept | student_t(3, 0, 2.5) |

*Linear regression* (Standardized change in warmth ~ Positive Demand + Negative Demand)

| **Parameter name** | **Prior** |
| --- | --- |
| b | flat |
| b_NegativeDemand | flat |
| b_PositiveDemand | flat |
| Intercept | student_t(3, 0, 2.5) |
| sigma | student_t(3, 0, 2.5) |

*Multinomial logit regression* (Belief Category ~ Positive Demand + Negative Demand)

| **Parameter name** | **Distributional parameter** | **Prior** |
| --- | --- | --- |
| b | muless | flat |
| b_NegativeDemand | muless | flat |
| b_PositiveDemand | muless | flat |
| b | mumore | flat |
| b_NegativeDemand | mumore | flat |
| b_PositiveDemand | mumore | flat |
| Intercept | muless | student_t(3, 0, 2.5) |
| Intercept | mumore | student_t(3, 0, 2.5) |

**Study 2**

**Moderation of Belief Manipulation by Attention**

We preregistered that we would check whether attention moderates the effect of condition on belief. In a binomial logit model controlling for attention, we do not find evidence for moderation by attention (all effects are non-significant, *p*s > .150). The model also exhibits a worse fit than the simpler model in the main text (AIC = 871 vs. 868).

**Study 3**

**Robustness Checks for Multinomial Tests of Participant Beliefs**

We preregistered that we would test the hypothesis that demand condition (Positive, Negative, Control) alters participants’ beliefs about the study’s hypothesis using two binomial logit regressions (one frequentist and one Bayesian). But there are three conditions in our experiment, including an active control where we tell participants that the intervention will not affect their ingroup warmth. Therefore, unlike in Studies 1 and 2, here we captured participants’ beliefs using a three-option multiple choice (increase warmth / decrease warmth / not affect attitudes). As a consequence, no single binomial logit model can test our hypotheses about participants’ beliefs. There are two analytical choices available for the frequentist (and Bayesian) models: use a single multinomial logit regression, or use three binomial logit regressions (one for each contrast). Any other choice would depart considerably from the analysis we preregistered or add unnecessary complexity.

In the main text, we reported multinomial logit regression results, as independent binomial logit regressions fail to account for any dependencies across contrasts. For transparency, and to ensure that any deviations from the preregistration do not alter the conservatism of our statistical tests, we report results from the independent binomial logit regressions here, in both frequentist and Bayesian frameworks. Results do not change from those reported in the main text:

1. A binomial logit regression shows that the Positive Demand condition significantly increased participants’ likelihood of believing the study’s hypothesis was that the intervention would **increase** ingroup warmth relative to control (B = 1.21, SE = 0.19, *z* = 6.38, *p* < .001), and relative to the Negative Demand condition (B = 1.52, SE = 0.19, *z* = 7.83, *p* < .001).
2. A binomial logit regression shows that the Negative Demand condition significantly increased participants’ likelihood of believing the study’s hypothesis was that the intervention would **decrease** ingroup warmth relative to control (B = 1.05, SE = 0.20, *z* = 5.33, *p* < .001), and relative to the Positive Demand condition (B = 2.34, SE = 0.27, *z* = 8.69, *p* < .001)
3. A binomial logit regression shows that the **control** condition significantly increased participants’ likelihood of believing the study’s hypothesis was that the intervention would **not change** ingroup warmth relative to the Positive Demand condition (B = 0.58, SE = 0.19, *z* = 3.10, *p* = 0.002), and to the Negative Demand condition (B = 0.72, SE = 0.19, *z* = 3.84, *p* < .001)

The same models in a Bayesian framework:

1. Bayesian binomial logit regression shows that the Positive Demand significantly increased participants’ likelihood of believing the study’s hypothesis was that the intervention would **increase** ingroup warmth relative to control (B = 1.21, 95% CrI = [0.85, 1.60], posterior probability [B > 0] > 0.999), and relative to the Negative Demand condition (B = 1.53, 95% CrI = [1.22, 1.84], posterior probability [B > 0] > 0.999).
2. Bayesian binomial logit regression shows that the Negative Demand significantly increased participants’ likelihood of believing the study’s hypothesis was that the intervention would **decrease** ingroup warmth relative to control (B = 1.06, 95% CrI = [0.73, 1.39], posterior probability [B > 0] > 0.999), and relative to the Positive Demand condition (B = 2.36, 95% CrI = [1.93, 2.83], posterior probability [B > 0] > 0.999).
3. Bayesian binomial logit regression shows that the Control condition significantly increased participants’ likelihood of believing the study’s hypothesis was that the intervention would **not change** ingroup warmth relative to the Positive Demand condition (B = 0.58, 95% CrI = [0.27, 0.9], posterior probability [B > 0] > 0.999), and relative to the Negative Demand condition (B = 0.73, 95% CrI = [0.42, 1.05], posterior probability [B > 0] > 0.999).

**Moderation of Belief Manipulation by Attention**

We preregistered that we would check whether attention moderates the effect of condition on belief. A multinomial logit regression controlling for attention shows that, among inattentive participants, both demand cues increased participants’ likelihood of believing the study hypothesis was that the images would decrease ingroup warmth (Positive Demand, B = 2.80, SE = 1.39, *z* = 2.02, *p* = .043; Negative Demand, B = 3.09, SE = 1.26, *z* = 2.45, *p* = .014). There was a significant, negative interaction of Positive Demand condition and attentiveness on believing the hypothesis was that the images would decrease ingroup warmth (Attention × Positive Demand: B = –3.79, SE = 1.42, *z* = –2.66, *p* = .008). Among inattentive participants, positive demand increased the likelihood of believing the study hypothesis was that the images would increase ingroup warmth (B = 2.04, SE = 0.97, *z* = 2.11, *p* = .035), but no other main effects or interactions were significant for this contrast (all *p*s > .270). In short, the beliefs of inattentive participants were also successfully manipulated by our intervention, though less cleanly than those of attentive participants. Importantly, both attentive and inattentive participants showed similar patterns of attitude updating (or lack thereof) across conditions. This is inconsistent with the possibility that demand affects a subgroup of participants, either attentive or inattentive.

**Mixture Model Analyses**

For completeness, we conducted mixture model analyses to identify potential latent classes of reactors and compliers in Study 3. We repeatedly fit mixture-of-Gaussian regressions predicting change in ingroup warmth as a function of condition assignment, with different numbers of mixture components (between *k =* 1 to *k =* 7 components. To mitigate instability, we required components to contain at least 4% of the sample. Overall, a two-component solution produced the lowest Bayesian Information Criterion (BIC) value. We therefore conducted bootstrap resampling (with 1,000 iterations) to calculate confidence intervals for each mixture component in this best-fitting model.

Inspecting this model, the two components represented 60.0% and 40.0% of the sample, respectively. Estimated slope coefficients suggest the first and second components showed no statistically significant evidence of demand effects, intervention effects, or reactance (Component 1: N = 450, B_PositiveDemand_ = 0.00, 95% CI [0.00, 1.64], B_NegativeDemand_ = 0.00, 95% CI [–1.82, 1.38], σ = 0.00; Component 2: N = 299, B_PositiveDemand_ = –0.25, 95% CI [–2.09, 1.85], B_NegativeDemand_ = –0.80, 95% CI [–3.02, 0.95], σ = 8.16). The extremely small value for Component 1’s residual standard deviation (σ) suggests even this best-fitting solution is effectively degenerate, inconsistent with the hypothesis that our data consist of a mixture of demand compliers and reactors.

**Subliminality of Intervention**

One might naturally wonder whether our intervention was truly subliminal. Images were presented sixteen times for approximately one frame (16 ms) across a small part of the screen (maximum 100-by-67 pixels, about 1° of visual angle at a typical viewing distance and with a typical computer monitor). Since our main aim was to characterize demand with an inert intervention, we can—and in the main text, do—directly test its inefficacy rather than rely on an argument from conscious imperceptibility. We therefore did not need to employ controls typically required in psychophysics (e.g., randomizing images across conditions, jittering inter-trial intervals) and instead chose to avoid deception (by keeping images condition-congruent).

But, as an additional check, we asked participants to pick which image they saw during their exit survey out of an array of 15 images. The array consisted of six true stimuli (i.e., Barack Obama and John McCain smiling, frowning, or with a neutral expression), as well as six politician distractors (i.e., Joe Biden and Donald Trump smiling, frowning, or with a neutral expression), and three unrelated distractors (a US flag, a dog, and a family at dinner). Table S2 reports the number of times participants identified each image as the intervention image by demand condition and by their political affiliation. We do not conduct a formal statistical test of accuracy. For this question, participants saw the whole image array at once. Also, by the exit survey, participants were aware they were in a study related somehow to political attitudes. These factors may have led them to form idiosyncratic beliefs about which image was most likely to have been presented. Indeed, many participants did pick a condition- and political orientation-congruent image. However, only 4.01% of participants (30 out of 749, about one in 20) correctly identified the picture they saw from the array of 15 images (between 0.9% and 7.6% in each subgroup). Strikingly, in every group, a plurality of participants picked a distractor image (US flag or dog; yellow highlights), and in every group, participants picked the distractor politician image (i.e., Biden or Trump) more often than the true stimulus image (i.e., Obama or McCain; blue-and-grey diagonal).

**Table S2.**

*Number of times each image was identified by participants as the intervention image during their exit survey.*

|  |  |  |  |  | **Liberal images** | | | | | | **Conservative images** | | | | | |  |
| --- | --- | --- | --- | --- | --- | --- | --- | --- | --- | --- | --- | --- | --- | --- | --- | --- | --- |
|  |  | **Unrelated distractor images** | | | **Positive** | | **Negative** | | **Neutral** | | **Positive** | | **Negative** | | **Neutral** | |  |
| **Politics** | **Demand** | **US Flag** | **Dog** | **Dinner** | **Obama smiling** | **Biden smiling** | **Obama frowning** | **Biden frowning** | **Obama neutral** | **Biden neutral** | **McCain smiling** | **Trump smiling** | **McCain frowning** | **Trump frowning** | **McCain neutral** | **Trump neutral** | **Correct %** |
| Cons. | 0 | 26 | 14 | 7 | 2 | 0 | 2 | 2 | 2 | 2 | 2 | 15 | 1 | 10 | 4 | 15 | 3.8% |
| Cons. | - | 37 | 10 | 4 | 1 | 0 | 2 | 1 | 1 | 5 | 1 | 8 | 1 | 17 | 0 | 22 | 0.9% |
| Cons. | + | 40 | 24 | 3 | 0 | 0 | 0 | 0 | 2 | 4 | 2 | 11 | 1 | 8 | 1 | 16 | 1.8% |
| Lib. | 0 | 36 | 25 | 5 | 5 | 4 | 7 | 2 | 6 | 23 | 3 | 4 | 4 | 6 | 2 | 8 | 4.3% |
| Lib. | - | 40 | 25 | 3 | 6 | 4 | 11 | 16 | 4 | 17 | 2 | 1 | 1 | 5 | 4 | 5 | 7.6% |
| Lib. | + | 29 | 35 | 7 | 6 | 17 | 1 | 4 | 8 | 25 | 0 | 2 | 0 | 2 | 2 | 1 | 4.3% |
| *Note:* Cell counts report the number of participants identifying they saw a given image during the intervention, when asked in the exit survey. We highlight in yellow the most frequently reported image among each subgroup of participants. Stimuli actually shown to participants are shaded in blue (i.e., political orientation- and condition-congruent images). Distractor images are shaded in grey. Participants could have formed theories about the nature of the subliminal image intervention, so we organize the table to contrast the true and distractor political orientation- and condition-congruent images. Cons. = Conservative; Lib. = Liberal; 0 = Control condition; - = Negative Demand condition; + = Positive Demand condition. | | | | | | | | | | | | | | | | | |

**Table S3.**

*Count of participants by experiment, condition, and reported belief about hypotheses.*

| **Experiment 1 – Dictator game** | **Positive demand** | **Control** | **Negative demand** |
| --- | --- | --- | --- |
| Reported belief about hypothesis: Large share | 141 | 35 | 18 |
| Reported belief about hypothesis: Small share | 108 | 214 | 234 |
| *% Large share* | *57%* | *14%* | *7%* |
| *% Correct* | *57%* |  | *93%* |
| *% Large different from chance, i.e., 50%?* | Yes, *p* = 0.04235 | Yes, *p* < 2e-16 | Yes, *p* < 2e-16 |
| *% Large different from Control condition base rate?* | Yes, *p* < 2e-16 |  | Yes, *p* = 0.001009 |

| **Experiment 2 – Moral vignette** | **Positive demand** | **Control** | **Negative demand** |
| --- | --- | --- | --- |
| Reported belief about hypothesis: Quit job | 213 | 152 | 79 |
| Reported belief about hypothesis: Not quit job | 38 | 99 | 171 |
| *% Quit* | *85%* | *61%* | *32%* |
| *% Correct* | *85%* |  | *68%* |
| *% Quit different from chance, i.e., 50%?* | Yes, *p* < 2e-16 | Yes, *p* = 0.0009911 | Yes, *p* = 5.88e-09 |
| *% Quit different from Control condition base rate?* | Yes, *p* < 2e-16 |  | Yes, *p* < 2e-16 |

| **Experiment 3 – Intervention** | **Positive demand** | **Control** | **Negative demand** |
| --- | --- | --- | --- |
| Reported belief about hypothesis: Warmer | 151 | 76 | 63 |
| Reported belief about hypothesis: No change | 81 | 112 | 74 |
| Reported belief about hypothesis: Colder | 19 | 56 | 117 |
| *% Warmer* | *60%* | *31%* | *25%* |
| *% Correct* | *60%* | *46%* | *46%* |
| *% Directionally wrong* | *8%* |  | *25%* |
| *% Correct different from chance, i.e., 33%?* | Yes, *p* < 2e-16 | Yes, *p* = 5.58e-05 | Yes, *p* = 2.56e-05 |
| *% Correct different from Control condition base rate?* | Yes, *p* < 2e-16 |  | Yes, *p* = 6.95e-16 |

Note: *p*-values from binomial tests, which are just another way of looking at the same likelihood as the logit regressions in the main text

**Supplemental Methods**

*Detailed View of Study Procedure*

1. After consenting, participants completed a brief **attention check** (same as used in de Quidt et al., 2018).


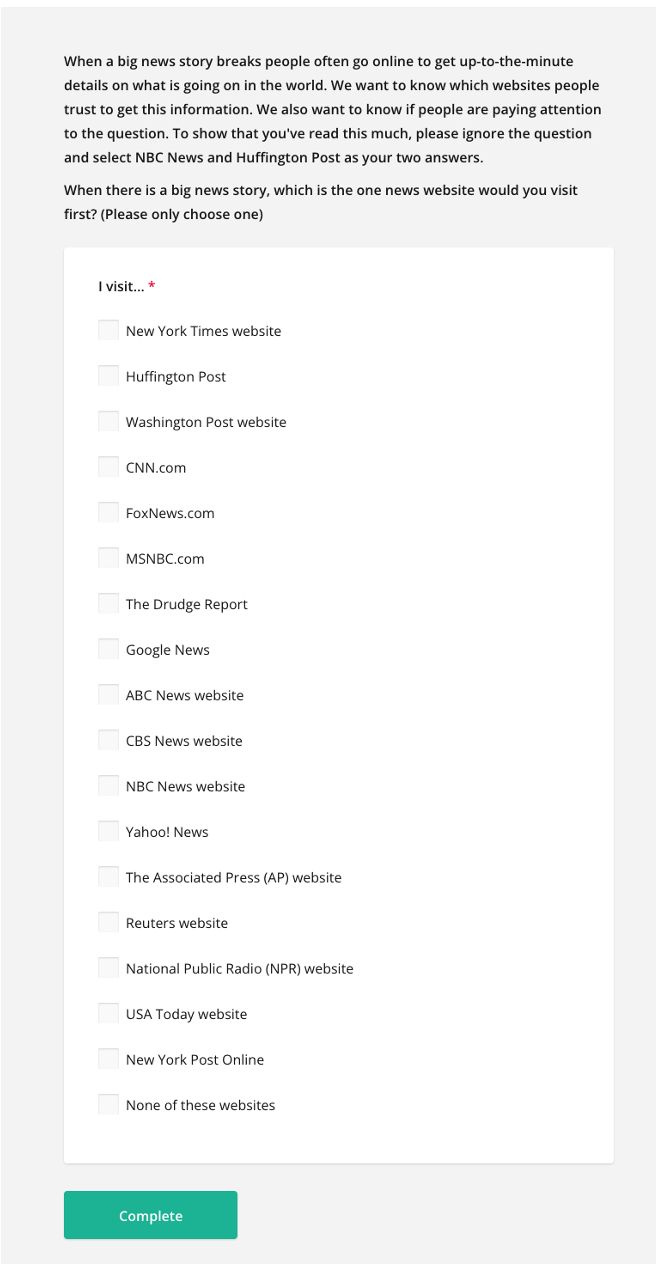


2. Participants filled out a one-item **political orientation survey**; this was used to define their political ingroup.


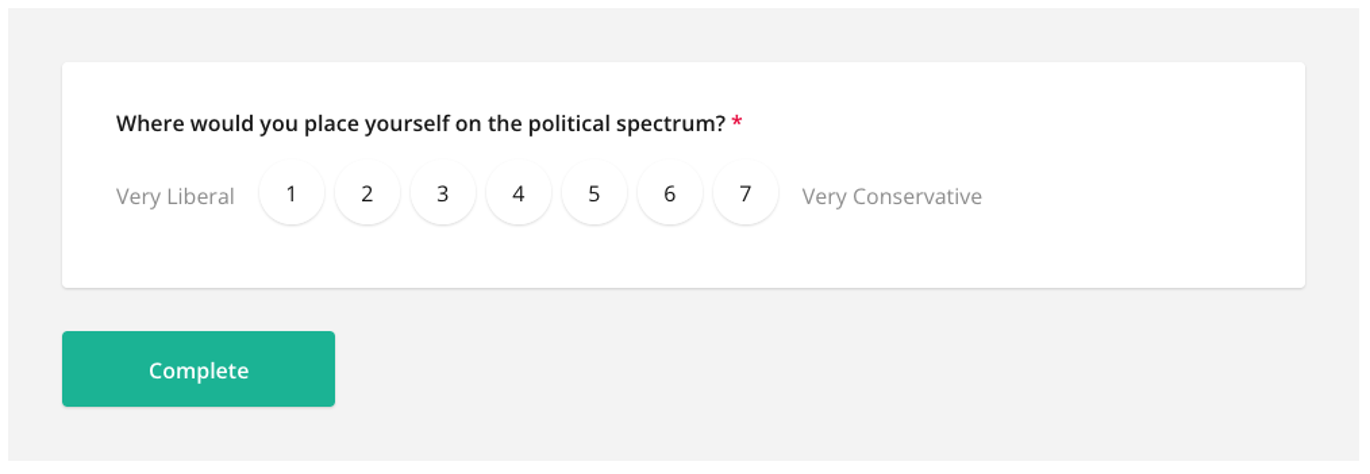


3. Participants completed a **pre-intervention feelings thermometer** measuring ingroup warmth. (here, we show the flow for a hypothetical participant who self-identifies as a conservative, >4 on the orientation scale above).


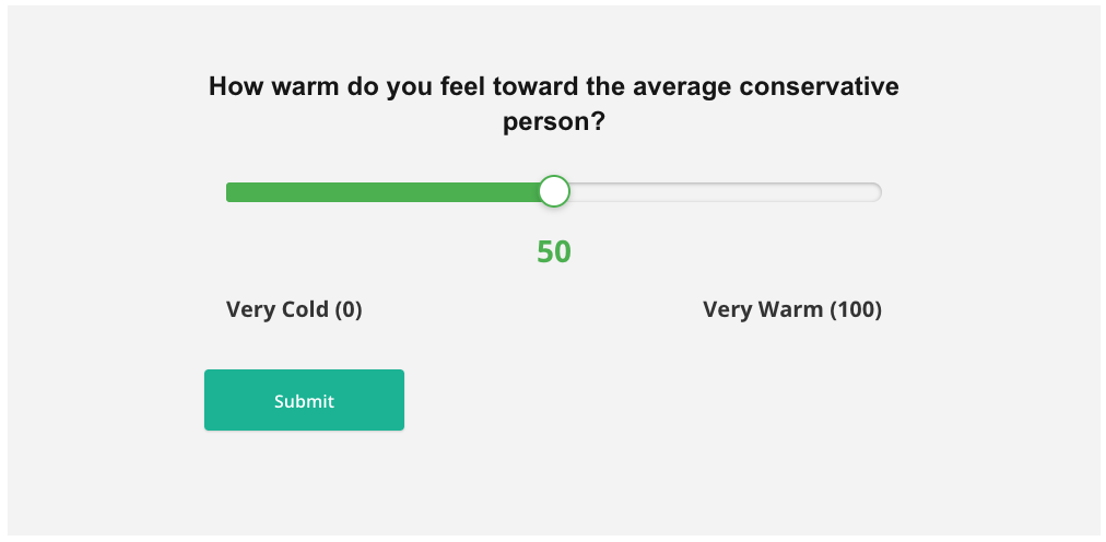


4. Participants received our experimental treatment – **positive demand, negative demand,** or a **neutral control**. (Here, we show the *positive* demand condition.)


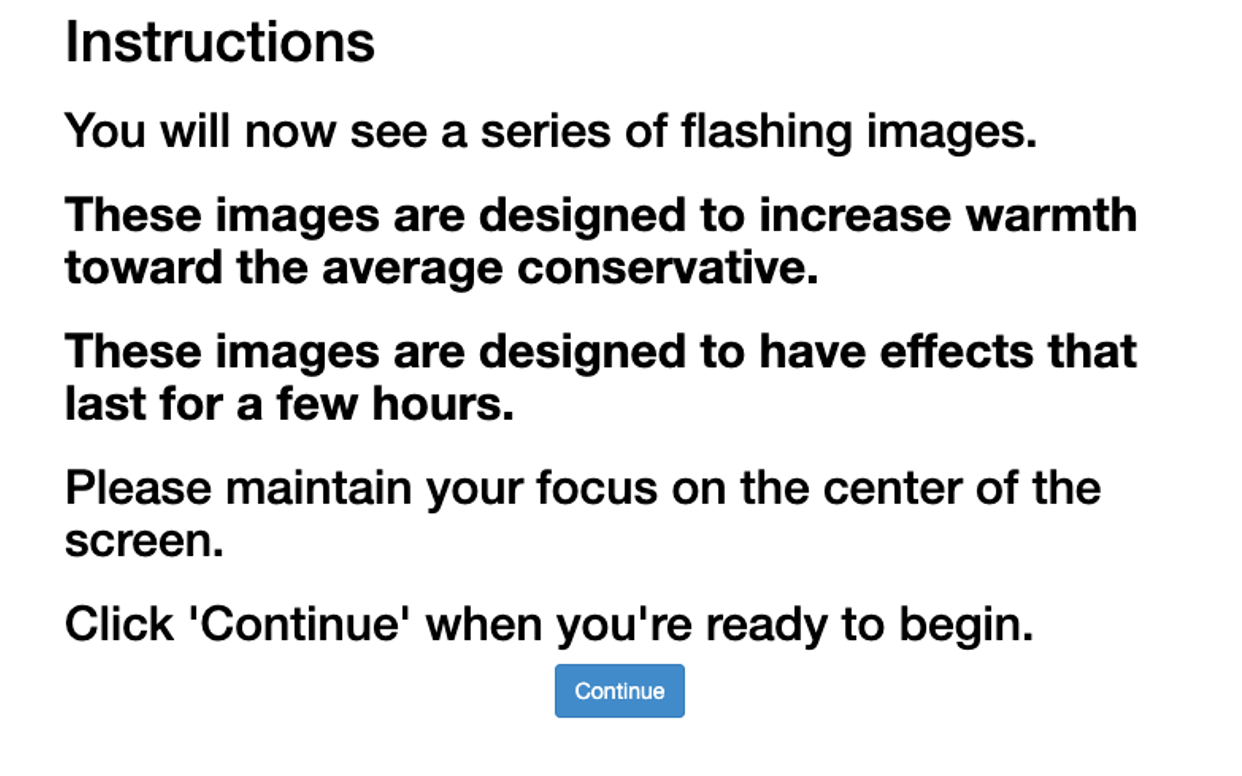


5. Participants saw our **inert intervention**. This consisted of 16 subliminal presentations of a condition- and ideology-congruent image. Each trial had the following flow:

1. Fixation cross (500ms; 100px by 100px, centered horizontally and vertically)


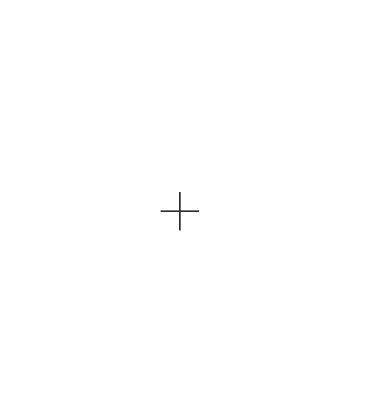


1. Congruent ingroup picture (16ms; 100px by 67px, inside a 600 px by 600px black rectangle, all centered horizontally and vertically)


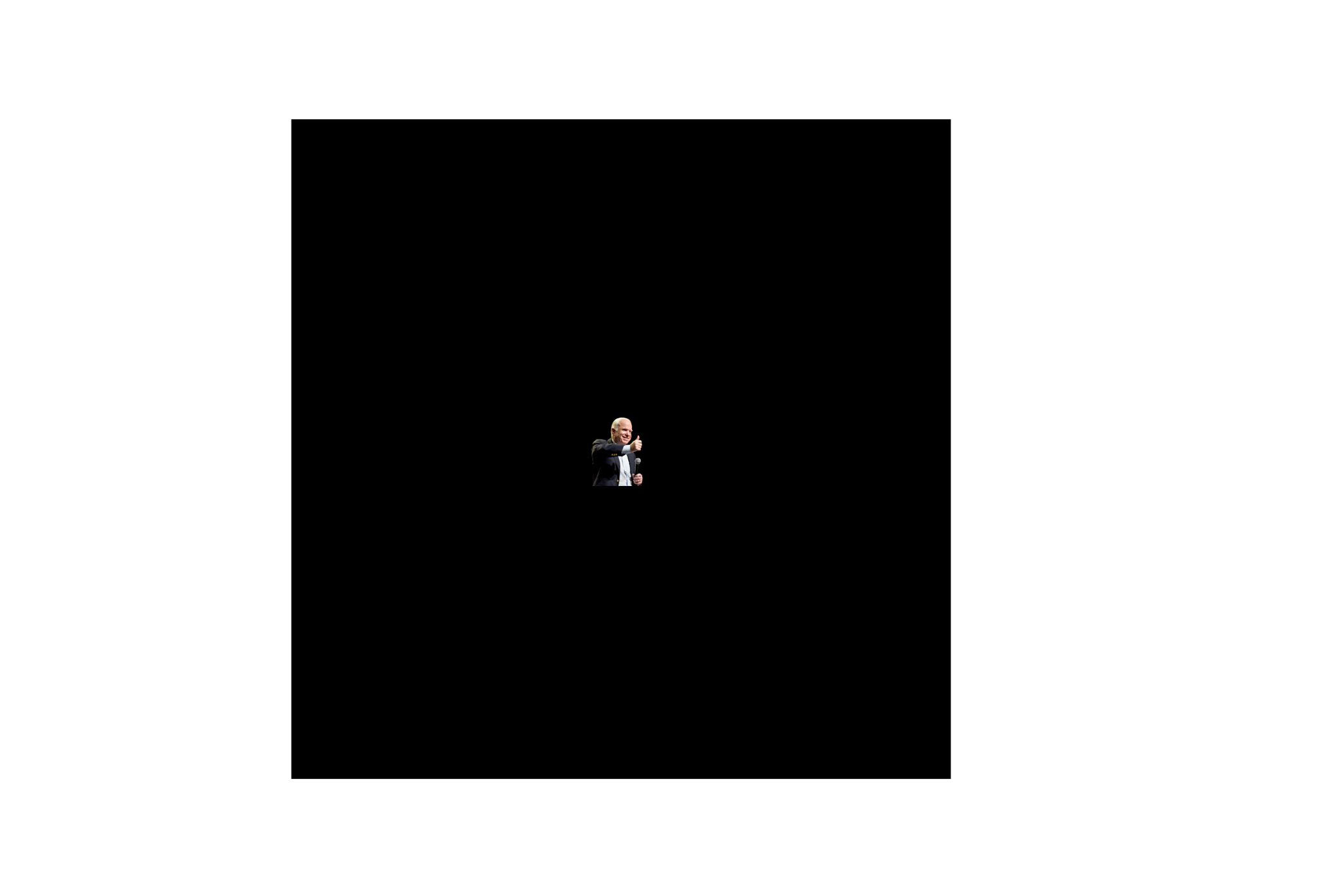


1. Ingroup ideology text (500ms; 48px font size, centered horizontally and vertically)


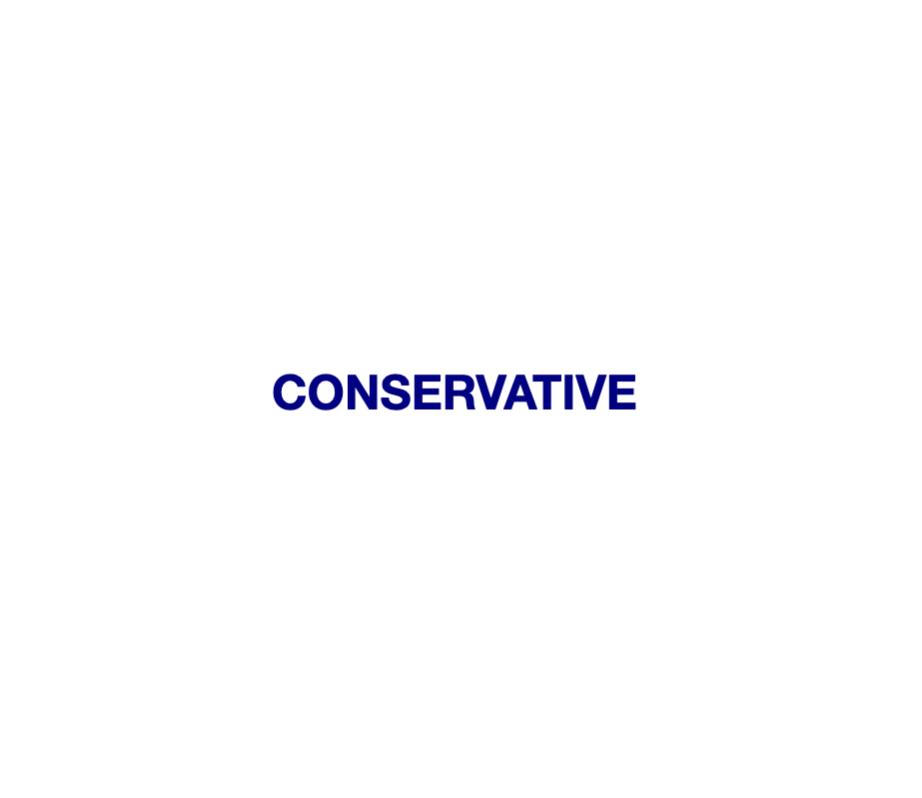


1. Repeat fixation cross (as in A)

6. Participants completed a **post-intervention feelings thermometer**. This was used to construct a change score, the dependent variable for our main analyses.


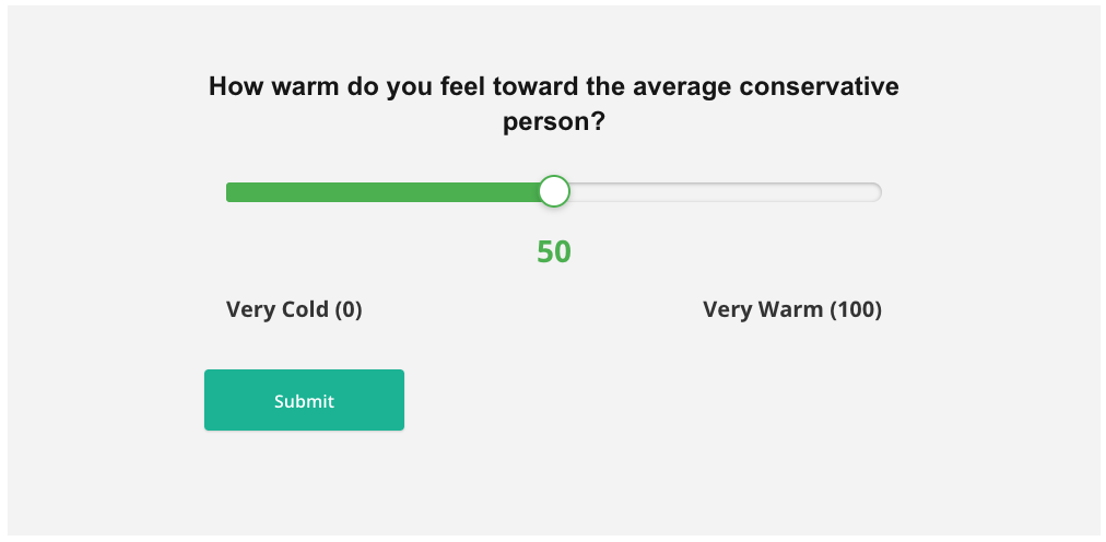


7. Participants completed an **exit survey** that captured their beliefs about the study’s hypothesis, after which they were debriefed.


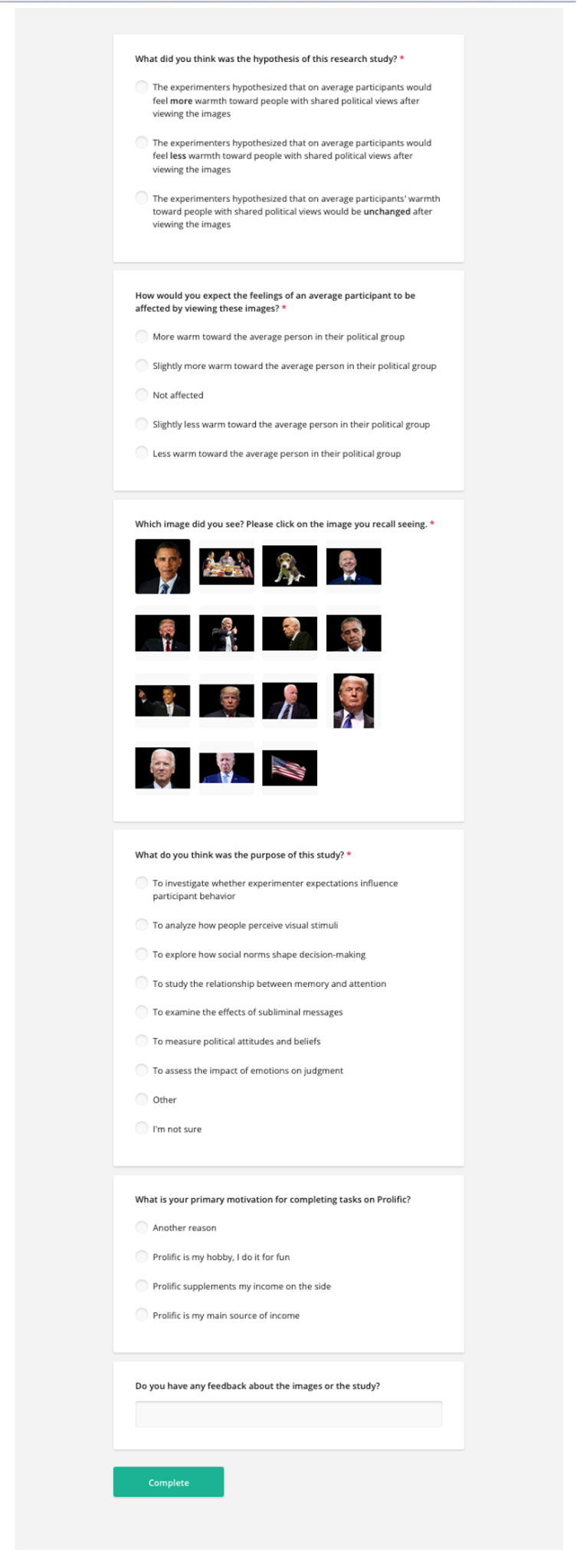

Supplement: Supplementary file 1 [file opmi-10-998-s001.docx]
